# TV vs. YouTube: TV Advertisements Capture More Visual Attention, Create More Positive Emotions and Have a Stronger Impact on Implicit Long-Term Memory

**DOI:** 10.3389/fpsyg.2019.00626

**Published:** 2019-03-21

**Authors:** David Weibel, Roman di Francesco, Roland Kopf, Samuel Fahrni, Adrian Brunner, Philipp Kronenberg, Janek S. Lobmaier, Thomas P. Reber, Fred W. Mast, Bartholomäus Wissmath

**Affiliations:** ^1^Department of Psychology, University of Bern, Bern, Switzerland; ^2^w hoch 2, Bern, Switzerland; ^3^Faculty of Psychology, Swiss Distance Learning University, Brig, Switzerland; ^4^Admeira, Bern, Switzerland; ^5^Department of Epileptology, University of Bonn, Bonn, Germany

**Keywords:** television, eye-tracking, skin-conductance response, long-term memory, implicit memory, advertisements, YouTube, purchase intent

## Abstract

In an experiment, effects of commercials that are either shown within a TV program or embedded in YouTube videos were compared. These two media environments have not yet been compared empirically in terms of their advertising impact. A within-subjects design and a multi-method approach were used (*N* = 36). Eye tracking data show that more attention is allocated to advertisements that appear within a TV program compared to the YouTube-condition and the viewing experience elicited more positive emotions in the TV-condition. Two days after reception, no difference in recognition, likeability, and purchase intention occurred, but in terms of implicit long-term memory: In the TV condition, brands that were previously advertised but no longer remembered elicited stronger skin conductance change than brands for which no advertisements had previously been shown. In terms of advertising impact, TV seems to still be the better choice for advertisers. Presentation mode should be considered in future evaluation of advertisement potential.

## Introduction

Media usage patterns have changed significantly over the past decade. The use of moving images outside the TV set is steadily increasing. Since its launch in 2005, YouTube provides moving images to a wide audience and has established itself as the most successful and most visited online video-sharing service (Snelson, 2011). An analysis by the European Trade Association for Marketers of Advertising (EGTA, 2018) shows that TV is currently still used more. This is particularly true when comparing the duration of TV consumption with that of YouTube consumption: Across all age groups, the study shows that 71% of total video time is spent on television, compared to only 6.4% spent on YouTube (EGTA, 2018). Yet, change is under way in particular among younger users. For example, current data show that American teenagers spend 34% of their total video time watching YouTube (Trendera, 2017). Another change concerns the so-called c: More and more media users use several devices simultaneously and watch TV and YouTube at the same time, for example.

This change in media usage behavior has also affected advertising. In the second half of the 20th century, television advertising was regarded as an indispensable communication tool between companies and customers with the power to convey and shape lifestyles and values (O’Barr, 2010). This has changed during the last two decades. Passive TV consumption has given way to more active consumption, which is increasingly making it possible to actively avoid advertising (e.g., O’Barr, 2010; Teixeira et al., 2010). This change was accompanied by various developments such as video recording devices, time-shifted television, the Internet, social media in general, and the emergence of YouTube in particular (Shin and Lin, 2016). Hence, while still being an important marketing element, TV spots may no longer be as decisive for advertising a product or a brand. This is also reflected in the fact that expenditure on TV advertising – even if a plateau has been reached – is still slightly rising. In 2017, $178 billion was spent worldwide on TV advertising. Digital ad spending (including YouTube) reached $209 billion worldwide, which is 41% of the market compared to 35% market share for TV advertising (Kafka and Molla, 2017). According to United States senior marketers, YouTube accounts for 27% of the digital ad spending (trends.e-strategyblog.com, 2018). Thus, the spending on TV advertising is still considerably higher than the spending on YouTube advertising, even though change is under way.

The goals of TV advertising are ultimately the same as those of YouTube advertising (cf. Dehghani et al., 2016). According to Martínez-Camino (2008), advertising is a way of communication that consists of an offer of information and a request for services. The aim is therefore to increase brand awareness and to inform about new products (e.g., Sutherland and Sylvester, 2000), which in turn should persuade the viewer to buy a product (cf. Martínez-Camino and Pérez-Saiz, 2012). Brand awareness and purchase intention are thus two central elements of advertisement success (cf. Venkatraman et al., 2015). In the past, various factors were identified as being relevant in moving image advertising in order to increase brand awareness and purchase intentions (e.g., Calder and Malthouse, 2008). According to Bronner and Neijens (2006), the following factors account for advertising effectiveness: information (does a commercial offer new information?), practical use (does a commercial provide useful advices to the viewer?), stimulation (does a commercial make the viewer curious?), negative emotions/irritation (does a commercial irritate the viewer?), transformation/entertainment (does a commercial induce enjoyment?). Dehghani et al. (2016) examined the influence of the factors information, entertainment, and irritation on the effectiveness of YouTube advertisement. As a further factor, they included customization. This factor relates to whether an ad meets the needs of a customer and is therefore linked to the “practical use” factor proposed by Bronner and Nijens. The findings of Dehghani et al. (2016) show that YouTube advertisement positively influences the costumers’ purchase intention. Alike studies concerning TV advertisement (e.g., Aaker and Bruzzone, 1985), all factors were identified as drivers of advertisement success. Among those, perceived entertainment and the customization of advertisements were found to have the strongest effect.

Changes in media usage behavior offer new ways to reach users for advertisers. At the same time, this means that it is essential to know which channel has which effect. The abovementioned results of Dehghani et al. (2016) suggest that the factors influencing YouTube advertising effectiveness may be quite similar to the factors affecting the effectiveness of TV advertisement. Also, existing research shows that TV advertising (e.g., Poels and Dewitte, 2006) as well as YouTube advertising (e.g., Dehghani et al., 2016) can positively influence the consumers’ brand awareness and purchase intention. However, to the best of our knowledge, there is currently no empirical data available comparing TV and YouTube in terms of their advertising potential. We claim that it would be important to clarify this issue, as it is crucial for marketers in order to decide which advertising platform is most appropriate. In the current study, we have therefore compared the effect of TV advertising with the effect of YouTube advertising in an experimental setting.

TV and YouTube contents are usually consumed on different devices. YouTube is mostly used on handheld devices; this is the conclusion of Ruffalo Noel Levitz (2012) as well as of a recent representative European media consumption study (Kopf, 2017). In accordance, Buzzetto-More (2014) found that students most likely visit YouTube from a mobile device: About two-thirds of the respondents indicated that they access YouTube using a handheld device. In contrast, TV content is still predominantly seen on a television set (e.g., Kopf, 2017). Moreover, television advertising consists of blocks of various commercials that are shown sequentially. YouTube advertisements are embedded in a different way. In contrast to TV, there is no blockwise presentation of advertisement on YouTube, but single commercial advertisements that are embedded between two YouTube clips. To ensure ecological validity, we use the prototypical TV setting and the prototypical YouTube setting in our study: We compare the effect of commercials that are shown sequentially within a TV program on a TV set (TV condition) with the effect of single commercials that are shown between separate You-Tube clips on a smartphone (YouTube condition).

We accomplished a within-subjects design. All participants watched advertisements in both conditions. Thus, possible confounds on the part of the subjects were ruled out. While we conducted an experiment, we still aimed to ensure ecological validity: Instead of presenting the stimuli in a laboratory, we placed participants on a sofa in a furnished apartment.

In order to comprehensively assess the advertising impact and in accordance with the proposals of Poels and Dewitte (2006), Micu and Plummer (2010), as well as Venkatraman et al. (2015) we have chosen a multi-method approach using a variety of indicators to measure advertisement effects. We assessed cognitive measures (recognition), eye movements, and physiological measures (skin conductance). Furthermore, we included established self-report measures that provide insights about subjective consumer attitudes and preferences which were previously shown to predict advertisement success (Poels and Dewitte, 2006; Smit et al., 2006; Venkatraman et al., 2015). Since, we aim to gain a deeper understanding of the underlying processes and the effects of the advertisements, we investigated short-term as well as long-term and implicit effects.

Initially, in a first session we focused on the reception phase. On the one hand, we have investigated whether there are differences between TV and YouTube advertisement in terms of attention during the reception phase. We did so by tracking eye movements (Venkatraman et al., 2009). According to Leven (2013) as well as Higgins et al. (2014), the percentage of fixations on an advertisement is a valid indicator of attention as well as engagement with this advertisement. On the other hand, we assessed the affect elicited by the advertisement: We examined whether the reception of the TV program or the YouTube program are judged as having a more positive emotions immediately after reception. Moreover, we assessed possible differences between the two conditions in how the advertisements were perceived.

Since existing research often neglects to scrutinize possible long-term effects of advertisements, we additionally assessed the advertising effect in a second session 2 days after seeing it. Recognition of the commercials was used as an indicator for brand awareness (cf. Venkataraman, 2007). Furthermore, we measured whether the advertisements presented 2 days before exerts an implicit effect on the participants. Even though self-report measures are popular and may predict effectiveness, they only assess those aspects that reach conscious awareness (Micu and Plummer, 2010). In contrast, implicit measures provide information about unconscious responses toward certain stimuli. We obtained skin conductance while participants viewed brand logos that belong to brands of which commercials were or were not seen during the reception phase 2 days earlier. Changes in skin conductance are a widely used marker for physiological arousal (e.g., Wang and Minor, 2008; Potter and Bolls, 2012) and have also been used as a physiological marker of implicit memory (e.g., klein Selle et al., 2018). Here, we measured changes in skin conductance as an implicit and psychophysiological indicator to assess whether the means and the embedded content on which the commercials were seen (YouTube condition or TV condition) has a modulating effect on the memory and recognition of the brand logos. Thus, the skin conductance change serves as an indicator of implicit long-term memory. Moreover, two self-report measures assessing attitude and purchase intention were obtained in Session 2: likability of advertised products and the willingness to buy the product.

Little is known about the mode of presentation of advertisement (blockwise vs. single presentation), but there is considerable amount of research showing that screen size matters: Existing studies show that a larger screen leads to more intense viewing experiences and more attention (e.g., Lombard et al., 1997), better memory (e.g., Grabe et al., 1999), more attention and persuasion (e.g., Reeves et al., 1999), and more immersive experiences (e.g., Hou et al., 2012). Due to these findings, we expect that the advertising effectiveness is generally higher in the TV condition than in the YouTube condition.

Specifically, we expect the following immediate effects: Compared to commercials shown in the YouTube condition, commercials shown in the TV condition lead to (1) increased attention in terms of fewer eye movements away from the screen, (1) more positive emotions, (3) and less subjective disturbance.

Furthermore, we expect the following longer-term effects: Compared to advertisements previously shown in YouTube conditions, commercials shown in the TV condition will be (1) better remembered and will (2) elicit stronger physiological responses. In addition, we expect (3) sympathy and (4) purchase intention to be higher for products previously advertised on TV compared to those that had been advertised on YouTube.

## Materials and Methods

### Participants

Thirty-six participants (15 males and 21 females) took part in the experiment. The average age was 24.20 years (*SD* = 5.07 years). Half of the participants were undergraduate students who received course credits for their participation. In order to enhance external validity, the other half of the participants were non-students. These participants were recruited from an existing database of potential participants as well as via social networks and they received 150 Swiss Francs (150 US$) for their participation. All participants are regularly watching TV and YouTube. All participants provided written informed consent. The study and protocol were reviewed and approved by the Ethics Committee of the Faculty of Human Sciences, University of Bern. The study is in line with the Code of Ethics of the World Medical Association, 1991 (Declaration of Helsinki). All participants were debriefed after the experiment.

### Design

#### Main Experiment

We used a one factorial multivariate within-subjects design. The independent variable was the medium in which the advertisement was presented (*TV condition* vs. *YouTube condition*): Each Participant watched several commercials embedded in a TV program as well as embedded in YouTube videos. The TV program was shown on a TV set (55 inches), the YouTube video on a smartphone (Samsung Galaxy S7, 5.10 inches). The order of the conditions was counterbalanced. The advertising effect was assessed using a multi-method approach: The dependent variables were eye movements, positive emotions, subjective disturbance by advertisements, recognition, skin conductance change, the attitude toward the advertised products in terms of likability, as well as purchase intention. These variables were assessed either at Session 1 during viewing (eye movements), shortly after viewing (positive emotions; subjective disturbance by advertisements), or 2 days after viewing (Session 2: recognition; skin conductance; attitude toward the product, likability).

#### Supplementary Experiment (Media Multitasking Setting)

In addition to the within comparison of the TV and the YouTube condition, a combined setting was accomplished: Participants were exposed simultaneously to both, a TV program on the TV set and a YouTube program on a smartphone. Eye movements were captured in order to examine which device attracts more attention. The supplementary experiment was carried out in order gain first insights on “media multitasking” (cf. Brasel and Gips, 2011).

### Stimulus Material

#### Main Experiment

Three TV programs and three YouTube video sets were prepared. All programs (TV and YouTube) lasted 20 min, 15 min of contents and 5 min of commercials. The TV programs were about (1) sports, (2) traveling or (3) animal documentary. Participants could choose one out of these three topics. The same topic was shown in both conditions (TV and YouTube). Original content by both public and private broadcasters was used. In contrast to the TV program that consisted of one feature story, the YouTube program consisted of seven original YouTube clips featuring the same topic as in the TV condition (sports, traveling, animal documentary).

Within both the TV and the YouTube program, 5 min of advertisement were presented in total. The advertisements were the same in both conditions and consisted of 11 (set A, cf. Table 1), respectively, 12 commercials (set B, cf. Table 1). Original commercials were used. Two sets of commercials were shown to the participants. Half of the participants watched set A within the TV program and set B within the YouTube program. The other half watched set B within the TV program and set A within the YouTube program. Since the commercials in set A were shorter, one more commercial was shown in set A than set B, thus keeping the total duration constant (5 min). Table 1 lists the commercials used for the study.

**Table 1:** Commercials presented in the TV as well as the YouTube condition.

Brand	Set A	Set B
Aldi	x	
Babbel	x	
Cafe Royal	x	
Galaxus	x	
Kuoni	x	
Mobiliar	x	
Raffaello	x	
Rivella	x	
Roviva	x	
Salt	x	
SBB	x	
Similasan	x	
Appenzeller		x
Migros		x
PostFinance		x
M&Ms		x
Zermatt		x
Vaudoise		x
Uncle Ben’s		x
Jack Wolfskin		x
Opel		x
Organspende		x
Ricola		x


In the TV condition, one block of advertisement was shown in the middle of the program. In the YouTube condition, the commercials were distributed across the whole program: After each clip a single commercial was shown.

#### Supplementary Experiment (Media Multitasking Setting)

For 5 min a TV program was shown on the TV set. At the same time, YouTube clips were shown on the smartphone. The TV program as well as the YouTube clips featured a topic that had not been chosen by the participant in the main experiment. Out of the remaining two topics one was randomly chosen. No advertisement was shown during the supplementary experiment. The following instruction was provided to the participants: “As the last part of the experiment, we will now show you two more short programs simultaneously on both devices. Your task is simply to watch these programs.” In order to avoid expectations, the instruction was deliberately kept short and openly formulated.

### Procedure

**FIGURE 1:**
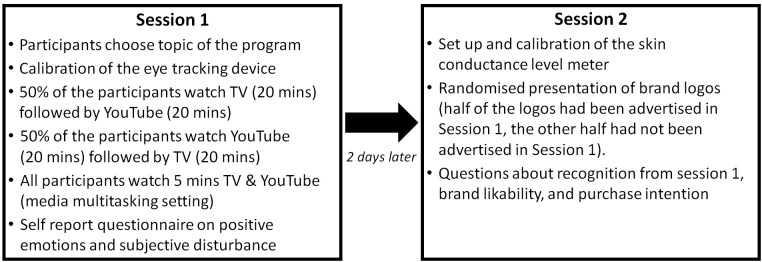
Summary of the procedure.

The procedure of the experiment is summarized in Figure 1.

The two parts of the experiment took part in two different locations. The first part (Session 1, reception phase) took place in a furnished apartment that was rented especially for the study in order to provide a real-life setting and to ensure ecological validity. We attempted to create a setting that is as close to reality as possible, while at the same time ensuring the necessary experimental control and standardization. In order to test to what extent a realistic setting could be provided, we asked the participants three questions about the situation (Q1: “During the experiment I felt comfortable,” Q2: “During the experiment I could relax,” Q3: “During the experiment I felt like in an experimental laboratory”). All questions were judged on a five-point Likert scale (1 = Strongly disagree; 5 = Strongly agree). The results revealed high values for the first two items (M_Q1_ = 4.03; M_Q2_ = 3.64) and low values for the third (reverse coded) item (M_Q3_ = 2.19). These results indicate that a realistic setting was achieved and that a higher ecological validity could be expected compared to a conventional laboratory study.

Participants were welcomed by the experimenter and asked to sit on a sofa. A mobile eye tracker was set up and calibrated. Participants were then told that they would watch both a TV and a YouTube program. They were also informed that they could choose from three different topics. In order to prevent expectancy effects, participants were told that the aim of the study was to examine whether different topics are received differently when presented on TV or on YouTube. It was not mentioned that we were primarily interested in the effects of the advertisements.

After the instructions, the two programs, respectively, video sets (duration of each program/video set including advertisements: 20 min) were shown on either a TV set (TV program) or on a smartphone (YouTube video sets) (*main experiment*). The two programs were on the same topics. Half of the participants watched the TV program first, the other half watched the YouTube video sets first. Eye movements were assessed during the presentation of the advertisements. After both conditions, participants were asked to fill out a questionnaire about how they felt during the program (positive emotions, disturbance by advertisements). At the end of the first part (Session 1), participants watched a TV program as well as a YouTube program for the duration of 5 min, while eye movements were captured (*Supplementary experiment, media multitasking setting*).

The second part of the experiment (Session 2, recognition phase) took place in a laboratory at the University of Bern 2 days after the reception phase. After being welcomed by the experimenter, a skin conductance level meter was set up. Participants then saw a set of brand logos. Half of these had been advertised in the commercials seen in Session 1, the other half had not been advertised in Session 1. For each logo, the participants had to decide whether they had seen a corresponding advertisement at Session 1. Participants also had to indicate for each logo how much they like the brand and whether they would buy the product. At the end of Session 2, the participants were thanked and debriefed.

### Measures

In order to comprehensively capture advertising success (cf. Venkatraman et al., 2015), a multi-method approach was chosen. Hence, various indicators were assessed: Besides traditional self-reported measures, objective and implicit data as well as cognitive measures were taken into account. Three variables were assessed during Session 1, four during Session 2 (2 days after the presentation).

#### Eye Movements (Session 1)

As described above, the measurement of eye movements is as a suitable indicator of attention (cf. Leven, 2013). In our experiment, the eye movements were recorded by a mobile eye movement camera type iView X (Sensomotoric Instruments, SMI). As an indicator of attention, we computed the percentage of fixations off the screen (i.e., the amount of time, the eyes are *not* focused on the commercial).

#### Positive Emotions (Session 1)

Numerous studies show that televised stimuli elicit emotional reactions (e.g., Schaefer et al., 2010; Weibel et al., 2011a,b). The Self-Report Emotion Inventory (Gross and Levenson, 1995) was developed to measure such reactions. We used the sub scale that assesses *positive emotions* (amusement, contentment, interest, and surprise) (sample item: “During viewing, I felt contentment”). These items have been used previously to assess the positive emotions of televised contents (e.g., Loertscher et al., 2016). For the subsequent analyses, the mean value was computed. Cronbach’s alpha turned out to be sufficient (0.74).

#### Subjective Disturbance by Advertisements (Session 1)

The subjective disturbance was measured with one single item: “While viewing the program I was disturbed by the advertisements.” (1 = *not at all*; 5 = *a lot*).

#### Recognition (Session 2)

In line with other studies (e.g., Venkataraman, 2007) we measured the brand awareness using a cognitive measure: We presented several brand logos and participants had to decide whether they had already seen the advertisements 2 days before or not. Then, participants had to press the “next” button to move on to the next logo. For the subsequent analyses, the percentage of correctly identified brands was calculated separately for the products shown in the TV and in the YouTube condition.

#### Skin Conductance Change (Session 2)

Skin conductance (SC) was assessed as an objective, valid, and reliable measure of unspecific emotional activity (cf. Boucsein, 1992). In the context of our study it serves as an indicator of implicit long-term memory. SC was continuously recorded during brand logo presentation using a PowerLab^®^8/35 system^1^, with a sampling rate of 20 Hz. Bipolar galvanic skin response finger electrodes (see footnote 1) were attached to the medial phalanx surfaces of the middle and index finger of the left hand using Velcro tape. This type of electrodes does not require the use of isotonic gel. Electrodes were attached 10–15 min prior to the experiment. As measure for skin conductance change, the difference between SC right after onset of the respective logo (baseline, see Guerra et al., 2012) and the SC peak within a period of 2 s after onset was taken into account.

#### Brand Likability (Session 2)

We measured the attitude in terms of likability with following question: “How much do you like this brand?” (1 = *not at all*; 5 = *a lot*). For the subsequent analyses, the mean value over all brands was computed separately for both conditions.

#### Purchase Intention (Session 2)

We measured the intention to buy the product by asking participants: “Which of the following statements is most appropriate for you” (1 = *I certainly would not buy the brand.*; 5 = *I could very well imagine buying this brand*). Again, the mean value over all brands was computed separately for both conditions.

## Results

### Main Experiment: Session 1

To test the effect of our manipulation (TV vs. YouTube), we first computed three comparisons for the variables assessed at Session 1 (i.e., the reception phase). Paired sample *t*-tests were carried out with the condition as independent variable, and fixations off screen, positive emotions, and disturbance as dependent variables. The percentage of fixations off the screen were significantly higher in the YouTube condition (*M* = 18.28, *SD* = 14.50) compared to the TV condition (*M* = 9.44, *SD* = 8.30), *t*(35) = 2.60, *p* < 0.05, *d* = 0.44. Furthermore, the mean rating for positive emotions was significantly higher for the TV condition (*M* = 3.53, *SD* = 0.74) compared to the YouTube condition (*M* = 3.08, *SD* = 0.94), *t*(35) = 2.94, *p* < 0.01, *d* = 0.50. Against our expectations, however, participants in the YouTube condition (*M* = 3.42, *SD* = 1.30) did not indicate that they felt more disturbed by the advertisements compared to the TV condition (*M* = 3.25, *SD* = 1.03), *t*(35) = 0.71, *p* = 0.48, *d* = 0.12.

### Main Experiment: Session 2

For Session 2 (recognition phase), we first tested whether there are differences between the brands shown in the TV Condition in Session 1 and those shown in the YouTube Condition. A paired sample *t*-test shows, that the recognition rate (percentage of correctly identified brands) did not differ between the TV condition (*M* = 0.69, *SD* = 0.16) and the YouTube condition (*M* = 0.71, *SD* = 0.18), *t*(35) = -0.53, *p* = 0.60, *d* = 0.09. In addition to recognition rates, we used signal detection theory to assess the sensitivity of the recognition. This ensures that the participants recognize the marks instead of just guessing (cf. Peters and Leshner, 2013). We calculated *A* prime, the percentage of hits minus percentage of false alarms (cf. MacMillan and Creelman, 2004). As with the recognition rate, *A’* did not differ between the TV condition (*M* = 0.52, *SD* = 0.18) and the YouTube Condition (*M* = 0.55, *SD* = 0.19), *t*(35) = -0.67, *p* = 0.51, *d* = 0.14.

We carried out a comparison (ANOVA) of skin conductance change between (1) brands that were correctly recognized, (2) brands that were shown in Session 1, but not recognized, and (3) brands that were not shown before. Five participants had to be excluded from the analysis of skin conductance change due to measurement errors caused by a malfunction of the skin conductance level meter. A comparison of the peaks of the skin conductance levels revealed a significant difference, *F*(2,122) = 3.38, *p* < 0.05. *Post hoc* comparisons (Tukey) showed that the psychophysiological reaction was stronger for brands that the participants had seen during Session 1, but did not recognize (*M* = 0.13, *SD* = 0.16) compared to brands that had *not* been seen during Session 1 (*M* = 0.06, *SD* = 0.06) (*p* < 0.05).

Further analyses of the skin conductance data show that the difference of skin conductance changes (1) between brands that were not recognized and (2) brands that had *not* been shown was significant for advertisements presented in the TV condition (*M*_notrecognized_ = 0.14, *SD* = 0.18; *M*_notshown_ = 0.06, *SD* = 0.06), *t*(35) = 2.12, *p* < 0.04, *d* = 0.36. However, the difference was *not* significant for advertisements presented in the YouTube condition (*M*_notrecognized_ = 0.11, *SD* = 0.15; *M*_notshown_ = 0.06, *SD* = 0.06), *t*(35) = 1.81, *p* = 0.08, *d* = 0.31.

No difference between the two conditions (TV vs. YouTube) occurred in terms of likeability (*M*_TV_ = 3.41, *SD* = 0.41; *M*_YouTube_ = 3.39, *SD* = 0.39), *t*(35) = 0.42, *p* = 0.67, *d* = 0.07, and purchase intention (*M*_TV_ = 3.60, *SD* = 0.38; *M*_YouTube_ = 3.64, *SD* = 0.39), *t*(35) = -0.58, *p* = 0.57, *d* = 0.10.

We then analyzed whether there are differences in likability and purchase intention between (1) those brands that were shown during Session 1 (either in the TV condition or the YouTube condition) and (2) brands that were *not* shown during Session 1. We found that brands that were shown during Session 1 were judged as being more likeable (*M* = 3.40, *SD* = 0.37) compared to brands that were not shown before (*M* = 3.15, *SD* = 0.41), *t*(35) = 5.58, *p* < 0.01, *d* = 0.64. Furthermore, the purchase intention was higher for brands that were shown during Session 1 (*M* = 3.62, *SD* = 0.34) compared to brands that were not shown before (*M* = 3.38, *SD* = 0.38), *t*(35) = 4.93, *p* < 0.01, *d* = 0.94.

### Supplementary Experiment (Media Multitasking Setting)

A paired sample *t*-test showed that participants fixated more on the TV set (*M* = 78%, *SD* = 17%) than on the smartphone (*M* = 22%, *SD* = 17%), *t*(35) = 9.41, *p* < 0.01, *d* = 3.29.

## Discussion

In our experiment, we compared advertisements presented on TV and YouTube. To our knowledge, this is the first empirical study directly comparing these two media environments. Our aim was to compare the prototypical YouTube setting with the prototypical TV setting and to gain initial insights into the question of the advertising effectiveness of YouTube compared to the advertising effectiveness of TV. Participants watched a TV program as well as a YouTube videos. We were interested in the influence of advertisements embedded in these two media and whether the two environments differ in their advertising effect. We compared the effect of commercials that are shown in a prototypical TV condition with commercials that are shown in a prototypical YouTube condition: The TV condition consisted of commercials that are shown sequentially within a TV program on a TV set, the YouTube condition consisted of single commercials that are shown between separate You-Tube clips on a smartphone. The effectiveness of the advertisements was captured using a multi-method approach. Apart from traditional self-report measures we assessed eye fixations while viewing the videos, recognition rates of the products that were advertised, and changes in skin conductance while seeing design logos. We examined both immediate and longer-term effects.

The analysis of the immediate effect show an increased number of eye movements off the screen during the advertisements shown in the YouTube condition when compared to the TV condition. This is in line with our hypothesis. Thus, in the TV condition more attention was allocated to the screen than in the YouTube condition. We suggest that this is on part due to the screen size. Previous studies have shown a positive correlation between attention and screen size (Lombard et al., 1997). In a supplementary experiment, participants were shown a TV program and a YouTube program at the same time. This data also shows that the TV attracts much more attention than the smartphone. These findings suggest that TV advertising has an advantage over YouTube advertising since more attentional resources with fewer distractions are directed to TV than to YouTube contents.

Furthermore, against our expectations participants did not feel more disturbed by commercials shown in the YouTube condition. However, in line with our hypothesis, the emotions of TV advertisements were rated as being more positive than advertisements on YouTube even though the advertisements were the same in both conditions. The reason could be that the blockwise presentation in the TV condition is more pleasant because it allows to easily switch from the content to some kind of advertisement mode and then back to the content mode. The single spot presentations in the YouTube conditions may feel more intrusive in comparison, which could be accompanied by a less positive feeling. However, this is speculation since we did not directly capture this issue.

The long-term effects were assessed 2 days after the presentation of the advertisement. The findings clearly show that video advertising has a positive impact: Brands that had been advertised in commercials previously were rated more positively than comparable brands which had not been advertised. Ratings on likability as well as on purchase intention were substantially higher for previously presented advertisements. This in line with various studies providing empirical evidence for TV advertising success (e.g., Lodish et al., 1995; Riskey, 1997; Hu et al., 2007; Rubinson, 2009). However, it should be noted that against our expectations, there has been no difference between TV and YouTube advertising in terms of likeability or purchase intent. Thus, the more positive emotions for the TV advertisements right after the reception did persist over time.

We show that the recognition rate was at 70% on average. This is also an indicator that video advertising has a positive effect (Venkataraman, 2007). However, against our hypothesis, in our experiment it made no difference whether the commercials were shown in the TV or in the YouTube condition. This means that the higher attention during the reception does not necessarily lead to better recognition. It may be that not that much attention is needed in order to remember a certain brand. Another reason could be that due to the ceiling effect the variance within the conditions was too low to observe an effect. The consideration of basic research on learning and memory could also help to explain this finding. It is possible that the arrangement of spots in the YouTube condition (single instead of blockwise) is an advantage: Primacy and recency effects can cause memory to be stronger for items presented first or last in a sequence compared to items presented in the middle of a sequence (Murdock, 1962). Thus, in a block of 11 or 12 spots – as was the case in our experiment in the TV condition – 9 or 10 spots appear in the middle of a sequence. Therefore, single spots may have led to better memory since they do not appear within a sequence, but stand alone between two YouTube clips. Furthermore, first information is better remembered when event-boundaries are distinctive and when the message is delivered in close temporal proximity to the event-boundaries (e.g., Swallow et al., 2009). In the context of our experiment, it could have been that single spots were perceived as sufficiently strong event boundaries so that they yielded better memory due to considerations similar to the serial position effect: a spot receives high priority in memory when it is presented near event-boundary, what is the case with all single spots. It could be that the advantage resulting from the single presentation mode has compensated for the weaker attention in the YouTube condition. How exactly serial position and event-segmentation interact in the context of advertisement presentations, however, remains open to future research. Future research will be necessary to address these topics.

Another interesting result revealed the analysis of the skin conductance data. One would expect that advertising that cannot be remembered will not have any effect. However, according to our results, this does not seem to be the case: the presentation of brands that were previously advertised, but were no longer remembered, led to a stronger psychophysical reaction than brands for which the participants did not see an advertisement. Looking at this comparison separately for TV advertising and YouTube advertising, we find this effect is only for TV advertising. This suggest that TV advertising has an effect on implicit long-term memory. Thus, advertisements are unconsciously remembered even though conscious access is missing. This could in turn have an impact on purchasing decisions, for example. Future studies will have to investigate as to whether skin conductance can predict actual behavior.

Our study has some limitations. Although internal validity is given due to the experimental setting and ecological validity due to the realistic scenario of reception. Furthermore, our design compares a prototypical TV setting with a prototypical YouTube setting. However, the two conditions differ in terms of screen size (small vs. big), device (TV set vs. smartphone), as well as mode of advertising presentation (blockwise vs. single spots). It is therefore not possible to fully determine which of the possible factors can account for the differences. Since there are no empirical studies on this topic yet, it was our goal to gain initial insights into the advertising effectiveness of YouTube compared to TV. As a consequence, we can only make statements about the effect of these prototypical settings. It would be interesting to add a YouTube desktop condition or a TV smartphone condition as an extension. This would allow finding out more about what causes the effect and what the role of screen size is exactly. However, this goes beyond our research question and would be an extension of our study that should be addressed in a further experiment. Future research will could also help to better understand the relationship between immediate advertising effects and long-term effects, including the role of the unconscious psychophysiological processes. A further limitation concerns the age of the participants. Younger users in particular prefer YouTube (e.g., Trendera, 2017). Therefore, age could be an important factor, and an analysis comparing older with younger participants would be interesting. In our sample, however, all participants, with one exception, were between 19 and 32 years old. The external validity is therefore only partially given, and an analysis including the age is not meaningful with our data. It would be useful to include older people in future studies and to investigate the influence of age.

## Conclusion

Our findings indicate that TV advertising has a stronger immediate impact on the recipient than YouTube advertising: It leads to more attention and to more positive emotions. In the longer term, this effect does not turn out on a conscious level, but is still effective on an unconscious level. We therefore conclude that TV advertising is still the better choice for advertisers, at least as long as the coverage of YouTube does not exceed that of classic television. The findings of our study have significant implications for advertising campaigns: Our findings suggest that in addition to coverage (reach), contact quantity (OTS) and time use, the mode of presentation (size and type of stimulus) needs to be considered as a criterion for effectiveness of advertising media. However, the distinction between conscious and unconscious effects makes the topic of effect quantification more complex, thus making it necessary to conduct more research.

## Data Availability

The datasets generated for this study are available on request to the corresponding author.

## Author Contributions

The original idea was conceived by RK and SF, with inputs regarding implementation and experimental design from RdF, BW, DW, JL, and FM. RdF and AB recruited the sample and carried out the experiments. The data analysis was carried out by PK and TR with inputs from BW, JL, RdF, and DW. DW wrote the manuscript with support from JL, FM, BW, and SF.

## Conflict of Interest Statement

Two of the authors (RK and SF) work for a marketing company (Admeira) that is issues advertisements in print, radio, TV and online domains such as YouTube, or web-presences of traditional media. Four of the authors (DW, RdF, PK, and BW) are employed by the company w hoch 2, a research office. The remaining authors declare that the research was conducted in the absence of any commercial or financial relationships that could be construed as a potential conflict of interest.

## Footnotes

1 www.adinstruments.com
